# Editorial on Genetics and Breeding of Polyploid Plants

**DOI:** 10.3390/genes16060662

**Published:** 2025-05-29

**Authors:** Eric Javier Martínez, Ana Isabel Honfi

**Affiliations:** 1 Laboratorio de Genética y Mejoramiento de Especies Forrajeras, Instituto de Botánica del Nordeste (CONICET-UNNE), Facultad de Ciencias Agrarias, Universidad Nacional del Nordeste (FCA-UNNE), Corrientes 3400, Argentina; 2 Programa de Estudios Florísticos y Genética Vegetal, Instituto de Biología Subtropical (CONICET-UNaM), Facultad de Ciencias Exactas, Químicas y Naturales, Universidad Nacional de Misiones (FCEQyN-UNaM), Posadas 3300, Argentina; ana.honfi@unam.edu.ar

## Editorial

Among plants, where evolutionary changes unfold across millennia and innovations are etched into the genome in silence, polyploidy stands as one of the most transformative forces known to science. Defined by the presence of more than two complete sets of chromosomes, polyploidy is no mere genetic glitch, it is a creative upheaval, a profound genomic reset that has shaped the course of plant evolution with both subtlety and spectacle.

Genome doubling is rarely a quiet affair. Instead, it launches a cascade of genetic and epigenetic shifts, chromosomal rearrangements, aneuploidy, gene loss, altered chromatin states, and dosage effects, like tectonic plates grinding into new configurations. These changes, far from being purely abstract, translate into tangible biological traits: enhanced vigour, altered fertility, bigger organs, increased plasticity, and even an uncanny ability to conquer new ecological frontiers. In short, polyploidy redefines what it means to be a plant.

Unsurprisingly, this phenomenon has captured the full attention of plant breeders. Many of our most important crops are polyploids, and their genetic complexity is both a challenge and an opportunity. The deliberate induction of polyploidy in wild relatives is now a critical step in unlocking new traits, improving resilience, and expanding the boundaries of what agriculture can achieve.

In this Special Issue, we explore the full spectrum of polyploidy’s impact, from its mechanistic underpinnings to its evolutionary consequences. Suppa et al. [1] tackled the practical challenge of inducing autotetraploidy in wild Arachis species, relatives of the cultivated peanut, through the careful application of mitotic inhibitors. Their refined colchicine seed treatment not only generated stable tetraploids, but also delivered the hallmark features of polyploidy: larger leaves and flowers, and with them, new breeding possibilities.

Understanding what happens after polyploidy is just as crucial. Honfi et al. [2] dissected the genomic architecture of diploid and tetraploid cytotypes in *Paspalum notatum*, revealing evidence of autopolyploid origin with minor structural rearrangements and genome downsizing, a kind of post-duplication detox, where excess is pruned back to function.

Of course, the allure of polyploidy often lies in its promise of heterosis, that mysterious boost in vigour seen in hybrids. Dudits et al. [3] examined this in triploid *Salix* hybrids, finding dramatic root growth and biomass increases. Behind this exuberance lay a symphony of hormonal changes and enlarged parenchyma cells, a physiological remix orchestrated by polyploidy itself.

Disentangling the pure effects of ploidy from genotype is no trivial task. Wijnen et al. [4] cleverly used haploid-inducer lines in *Arabidopsis thaliana* to create mapping populations phenotyped at both the monoploid and diploid levels. Their findings revealed not only ploidy-specific QTLs, but also complex genotype–ploidy interactions, which is proof that context is everything, even for genes.

For established polyploids like peanut, mapping complex traits remains a formidable but essential endeavour. Joshi et al. [5] constructed a high-density SNP linkage map in a tetraploid *Arachis hypogaea* population, identifying major QTLs for seed weight and shelling percentage. Interestingly, they found that epistatic interactions, those shadowy conversations between genes, often played a bigger role than the genes themselves.

Polyploidy also reshapes reproduction. Schedler et al. [6] examined four *Paspalum* species and discovered unexpected diversity: some exhibited traces of apomixis, others showed self-incompatibility or self-compatibility, and all reflected divergent evolutionary paths since polyploidization. Apparently, duplicating a genome also opens up new negotiations with nature’s reproductive script.

But in polyploidy, as in life, nothing comes easy. Mahmood et al. [7] tested the myBaits sequencing platform in hexaploid oats and found that while short-read data looked promising, validation told another story. Their results serve as a cautionary tale: in complex genomes, what you see is not always what you get.

Finally, Fan et al. [8] delved into the cytoplasmic–nuclear interactions in wheat, reminding us that in allopolyploids, the nucleus and cytoplasm must dance in synchrony—or stumble. Their exploration of *Aegilops kotschyi* cytoplasm effects showed impacts far beyond male sterility: seed size, growth rate, and even fertilization dynamics were reshaped, hinting at a deeper, more intricate genomic dialogue.

Together, these studies illuminate the multi-layered reality of polyploidy. It is both a driver of biodiversity and a tool for breeding, a force of disruption and a source of resilience. Its effects ripple through genomes, cells, organs, and ecosystems. And while we have come far in understanding it, polyploidy remains, at heart, a beautifully orchestrated mystery: part science, part serendipity.

This collection invites us not just to marvel at what polyploidy can do, but to think more deeply about how organisms rewrite their own blueprints in response to evolutionary pressures. The next time you walk past a stalk of wheat or a willow tree, remember, beneath their quiet surfaces lies the story of genomes bold enough to double down.
